# Retraction Note: A novel chalcone derivative S17 induces apoptosis through ROS dependent DR5 up-regulation in gastric cancer cells

**DOI:** 10.1038/s41598-026-35488-4

**Published:** 2026-01-19

**Authors:** Saiyang Zhang, Tingyu Li, Li Zhang, Xiangyu Wang, Hangqi Dong, Lili Li, Dongjun Fu, Yongchun Li, Xiaolin Zi, Hong-Min Liu, Yanbing Zhang, Hongde Xu, Cheng-Yun Jin

**Affiliations:** 1 School of Pharmaceutical Sciences, Key Laboratory of State Ministry of Education, Key Laboratory of Henan province for Drug Quality Control and Evaluation, Collaborative Innovation Center of New Drug Research and Safety Evaluation, Zhengzhou University, 100 Kexue Avenue, Zhengzhou, Henan 450001 China; 2 School of Basic Medicinal Science, Zhengzhou University, 100 Kexue Avenue, Zhengzhou, Henan 450001 China; 3 Departments of Urology, Pharmacology and Pharmaceutical Sciences, University of California, Irvine, Orange, USA

Retraction of: *Scientific Reports* 10.1038/s41598-017-10400-3, published online 29 August 2017

The Editors have retracted this Article.

After publication, concerns were raised regarding the data presented. Specifically, Figure 2D panel 1 and panel 3 appear to overlap. The Editors requested the original data underlying the study, but the Authors were not able to provide it. Additional concern was also identified in that the cell lines used in the study were contaminated with HeLa cells, and therefore not suitable model of a gastric cancer. The Editors therefore no longer have confidence in the data presented in the Article.

Saiyang Zhang agrees with the retraction. The Editors were not able to find current email addresses for Tingyu Li, Li Zhang, Yongchun Li. Remaining Authors did not respond to the correspondence about the retraction.

